# Prion Protein-Specific Antibodies that Detect Multiple TSE Agents with High Sensitivity

**DOI:** 10.1371/journal.pone.0091143

**Published:** 2014-03-07

**Authors:** Sandra McCutcheon, Jan P. M. Langeveld, Boon Chin Tan, Andrew C. Gill, Christopher de Wolf, Stuart Martin, Lorenzo Gonzalez, James Alibhai, A. Richard Alejo Blanco, Lauren Campbell, Nora Hunter, E. Fiona Houston

**Affiliations:** 1 Neurobiology Division, The Roslin Institute and Royal (Dick) School of Veterinary Sciences, Easter Bush, Edinburgh, Scotland, United Kingdom; 2 Central Veterinary Institute of Wageningen UR, Lelystad, The Netherlands; 3 Animal Health and Veterinary Laboratories Agency, Lasswade Laboratory, Edinburgh, Scotland, United Kingdom; Rocky Mountain Laboratories, NIAID, NIH, United States of America

## Abstract

This paper describes the generation, characterisation and potential applications of a panel of novel anti-prion protein monoclonal antibodies (mAbs). The mAbs were generated by immunising PRNP null mice, using a variety of regimes, with a truncated form of recombinant ovine prion protein spanning residues 94–233. Epitopes of specific antibodies were mapped using solid-phase Pepscan analysis and clustered to four distinct regions within the PrP molecule. We have demonstrated the utility of these antibodies by use of Western blotting and immunohistochemistry in tissues from a range of different species affected by transmissible spongiform encephalopathy (TSE). In comparative tests against extensively-used and widely-published, commercially available antibodies, similar or improved results can be obtained using these new mAbs, specifically in terms of sensitivity of detection. Since many of these antibodies recognise native PrP^C^, they could also be applied to a broad range of immunoassays such as flow cytometry, DELFIA analysis or immunoprecipitation. We are using these reagents to increase our understanding of TSE pathogenesis and for use in potential diagnostic screening assays.

## Introduction

Transmissible spongiform encephalopathies (TSEs) are a group of fatal neurodegenerative diseases that affect both animals and man and include bovine spongiform encephalopathy (BSE), scrapie and variant Creutzfeldt-Jakob disease (vCJD). Individuals affected with TSEs show long incubation periods before the onset of clinical signs. TSE infection is accompanied by the molecular conversion of a host-encoded glycoprotein, PrP^C^, into a diseased-associated aggregated isoform (PrP^Sc^, [1]); this isoform is partially resistant to proteolytic degradation and accumulates in the brain of infected individuals and often in peripheral tissues prior to neuroinvasion. Both PrP^C^ and PrP^Sc^ can be differentially glycosylated (at asparagine residues 184 and 200, ovine sequence), possess a single disulphide bond and carry a C-terminal glycosylphosphatidylinositol anchor; whilst PrP^C^ and PrP^Sc^ have the same primary structure, they differ both in their biochemical properties (such as solubility in detergents, resistance to proteolytic cleavage, denaturation with chaotropes i.e. guanidium) and secondary and tertiary structure. Following treatment with proteinase K (PK), different forms of PrP, which vary in relative molecular mass and result directly from differential cleavage events that are related to the strain of TSE agent, can be observed in animals and humans using both Western blotting and immunohistochemical approaches in an antibody-dependent manner [2]–[6]. In mammals, DNA encoding the open reading frame of PRNP is relatively well conserved and exhibits approximately 90% similarity across species [7]. Variation does, of course, exist in the PRNP gene within and between species. The most notable and well characterised examples of polymorphism (which are often associated with susceptibility to prion disease [8]–[10]) within an animal species are found in sheep and goats. In sheep, a 3-letter nomenclature is used to describe the most common alleles, where each letter denotes the amino acid encoded at the specific codon. A(alanine)_136_ R(arginine)_154_Q(glutamine)_171_ (ARQ) describes the wild type allele in sheep, but multiple variants have been identified such as the V (valine) RQ, ARR and ARH (where H is histidine) alleles [11], [12], as well as polymorphisms at other codons. Between species, variation in the primary sequence of PrP may elicit biological/functional effects, whereby, the outcome is naturally dependent on the change itself. For example, amino acid variation between codons 106–112 (human numbering) strongly influences the binding of the antibody 3F4 [13], [14].

A major aim of TSE research is the development of research tools that are applicable to multiple species, have utility in a wide range of immunoassays, have the potential to discriminate between PrP^Sc^ and PrP^C^ and/or may be used to develop diagnostic tests or therapeutic regimes. To date, this has been achieved, in part, by a number of academic research groups [15]–[21] and commercial companies with notable examples including, but not limited to, the anti-PrP antibodies P4 and L42 [22], 8H4 [23], KG9/FH11 and BG4 (The Roslin Institute, (http://www.roslin.ed.ac.uk/tseresourcecentre)), 6H4 and 15B3 [24], 3F4 [25], 12B2/94B4 and 100B3 (www.wageningenur.nl/prionantibody), T1 and T2 [20], The SAF/Sha (including Sha 31) and BAR-series of antibodies [26], the ‘R’-series of antibodies i.e. R145 [27], the POM monoclonals [28], the ‘ICSM’ antibodies [29].

Our interest in developing our own panel of prion antibodies was to replace commercially available antibodies, which at the time were expensive and did not provide in some cases the required sensitivity, for use as antigen capture and detector in sandwich immunoassays and other routine TSE confirmatory assays. We considered making new antibodies at a time when there was limited availability of a range of antibodies with cross reactivity to different species, especially sheep/ruminants and also a limited range of epitopes recognised. To generate a panel of ‘broad spectrum’ anti-PrP antibodies, we immunised PRNP^−/−^ null mice with an N-terminally truncated form (spanning residues 94–233) of recombinant ovine PrP (abbreviated as recOvPrP, 94–233). A truncated protein was used to avoid generating antibodies that the epitope would be in the N terminal region of PrP, which is cleaved following treatment with proteinase K (PK). We rationalised that the resultant antibodies would cross react with PrP of species other than sheep by virtue of the high degree of homology of PrP across mammalian species.

We report a unique collection of 28 new PrP antibodies and show the utility of many of these in Western blotting and IHC analyses of tissues from TSE-infected animals. Using Pepscan analyses, the epitopes of the antibodies have been located to 4 discrete regions within the central and C-terminal domains of the PrP molecule. Interestingly, not all of the antibodies were amenable to Pepscan mapping, suggesting that some may recognise conformational epitopes. The reagents have a broad species-specificity and many cross react with multiple TSE agents. In comparative analysis with commercially available prion antibodies, similar or better results can be achieved. We offer these new tools for collaborative projects designed to increase knowledge of TSE agents in natural and experimental systems, for the development of screening assays or diagnostic tests and for therapeutic intervention strategies.

## Materials and Methods

### Animals/tissues Used in this Study and Ethics Statement

All animal work was reviewed and approved by ethical review panel at The Institute for Animal Health and The Roslin Institute and conducted under the authority of Home Office. Animal tissues were obtained from a variety of sources (typically from ongoing studies from co-authors/collaborators) and as such are described in detail at specific sections throughout the manuscript.

### Expression, Purification and Refolding of Recombinant Ovine PrP (Residues 94–233, ARQ Allele)

For immunisation and *in-vitro* assays, a truncated form of recombinant ovine PrP spanning residues 94–233 (ovine numbering, recOvPrP, 94–233, ARQ allele) was prepared according to published methods [30]–[32]. Briefly, the protein was expressed from the pTrc plasmid, hence incorporates an N-terminal hexa-histidine tag, in 1B392 *Escherichia coli* cells and protein-containing inclusions bodies were isolated by centrifugation after enzymatic lysis. Protein was solubilised in a urea-containing buffer and was purified, under reducing conditions, by sequential chromatography steps based on immobilised nickel ion affinity and cation exchange resin. After overnight oxidation of the disulphide bond by copper ion catalysed oxidation, the urea was removed by dialysis into 50 mM sodium acetate, pH5.5. The final protein solution was adjusted to a final concentration of 0.5 mg/ml in phosphate buffered saline (PBS), pH 7.2, immediately before use in immunisations. For Western blotting assays, recPrP from other species were produced as described [31], [33] and following the same generic protocol as given above. For the remainder of the work, and unless specified, the numbering of amino acids for native or recombinant PrP corresponds to that represented by the ovine sequence.

### Immunisation Strategy

Nine young adult, PRNP^−/−^ mice (129 Ola, all male [34]) were assigned to 3 separate experimental groups each consisting of 3 mice. Each experimental group was subject to a specific immunisation regime (Table S1). Group 1 mice were immunised subcutaneously with 40 µg recOvPrP, 94–233 emulsified in an equal volume of Titre max (Stratech). Group 2 mice were immunised intraperitoneally (i/p) with 40 µg recOvPrP, 94–233 mixed with Quil A (1 µl added per 10 µg recOvPrP, 94–233 immunised, stock concentration = 2 mg/ml, Superfos, Denmark). Group 3 mice were immunised i/p with 40 µg recOvPrP, 94–233 emulsified with an equal volume of Alum (Pierce). Mice were boosted every 4, 6 or 8 weeks after the first immunisation (as shown in Table S1, for example mouse ID 0, in experimental group 1 was boosted every 4 weeks after the first immunisation) and until there was a marked difference in antibody titres compared to that seen in pre-immune sera (see section ‘Evaluation of antibody titres in tail blood using ELISA’). Typically, mice received between 3 and 6 boosts, after the 1^st^ immunisation. A pre-immune (control) serum was collected from each mouse 9 days before starting the immunisations. 5–7 days after each immunisation, blood was collected for testing of antibody response using an ELISA.

### Preparation of Anti-sera from Tail Blood Samples

Whole blood collected from mouse tails was incubated for 1 h at 37°C, to form a clot, then centrifuged (13,000 g using a bench top centrifuge). The supernatant was diluted 1∶10 using sterile PBS, aliquotted and frozen at −20°C until required.

### Evaluation of Antibody Titres in Tail Blood Using ELISA

Wells of a microtitre plate (Falcon) were coated with full-length recOvPrP spanning residues 25–233 (abbreviated as recOvPrP, 25–233) at a final concentration of 2 µg/ml diluted in carbonate coating buffer. Carbonate buffer was prepared by mixing 40 ml of 0.1 M Na_2_CO_3_ (pH 11) with 60 ml of 0.1 M NaHCO_3_ (pH 8). Plates were sealed and then incubated overnight at 4°C. The supernatant was decanted and wells washed twice using PBS containing 0.05% (v/v) Tween 20 (PBS-T, Sigma). Wells were blocked by incubation with 200 µl of 5% (w/v) Marvel (low-fat milk powder) prepared in PBS-T (PBS-TM) for 2.5 h at 37°C and then washed three times using PBS-T. Sera from tail blood samples were serially diluted in PBS-TM (1∶1000 to 1∶32,000) and applied to the wells in duplicate. Negative control wells were incubated with both PBS-TM alone and pre-immune sera samples (diluted 1∶500 in PBS-TM); positive control wells were incubated with the anti-PrP mAb FH11, which binds an epitope within the N-terminal domain of PrP (used at a final concentration of 1 µg/ml prepared in PBS-TM, obtained from the TSE RC). Samples were incubated for 1–3 h at 37°C and then washed three times using PBS-T. Secondary antibody (peroxidase conjugated, goat anti-mouse IgG, DAKO) was diluted 1∶5000 in PBS-TM and 100 µl applied to each well (with the exception of a substrate control well). The secondary antibody reaction was incubated for 1 h at 37°C. The plates were washed four times and then 100 µl ABTS solution (HRP substrate, Roche) added per well and incubated for approximately 20 min. The colorimetric readout from test and control samples was measured used a spectrophotometer at 415 nm.

### Fusions and Cloning

MAbs were prepared using conventional hybridoma technologies [35]. Briefly, 5–6 weeks after the final boost and 5 days prior to fusion, mice were injected with 50 µg recOvPrP, 94–233 intra-peritoneally with no adjuvant. Four days prior to fusion mice were injected with 50 µg recOvPrP, 94–233 (no adjuvant) intravenously. Spleens were removed, cell suspensions were fused with mouse myeloma cell line SP2/0 and hybrids were selected in hypoxanthine-aminopterin-thymidine (HAT) medium. Supernatants from cell cultures were screened by dissociation-enhanced lanthanide fluorescent immunoassay (DELFIA, see section Screening of culture supernatants using DELFIA and [36]), positive wells were selected (on the basis of giving highest reading compared to positive and negative controls) and cells were cloned by limiting dilution. Seven fusions were prepared resulting in the production of 28 antibodies in total (Table S1). The nomenclature of the mAbs correlates to the identification of the plate, row and well number of the final cloned antibody i.e. mAb BC6 originated from plate B, row C, well 6. Additionally, all antibodies are pre-fixed with the identification ’ROS’ indicating their derivation from the Roslin Institute i.e. ROS-BC6. The isotype of each mAb was determined using mouse monoclonal isotyping kits (Roche Isostrip or equivalent).

### Screening of Culture Supernatants Using DELFIA

Briefly, 96-well plates (Wallac) were coated with 0.2 µg/well purified recOvPrP, 25–233, overnight at 4°C. The supernatant was removed and wells were washed twice with PBS before blocking with 2% (w/v) BSA/PBS buffer, pH 7.2 for 1 h with agitation. 100 µl of culture fluid supernatant was added to each well for 1 h at room temperature with agitation. FH11 was applied to control wells at a concentration of 1 µg/ml. After 3 washes (PBS buffer) europium-labelled goat anti-mouse IgG (Dako) was diluted 1∶10,000 in assay buffer (Wallac) and incubated for 1 h at room temperature. After a final wash step, 200 µl of enhancement solution (Wallac) was added to each well and incubated for 10 min at room temperature with agitation. Plates were analysed using the Victor 1420 multi-label counter (Perkin Elmer/Wallac). Each clone’s specificity for both ovine recPrP and PK-resistant PrP^Sc^ was also tested in other immunoassays (including flow cytometry (not shown), Western blotting or immunohistochemistry (IHC)). Growth, freezing, thawing and expansion of cloned supernatants were conducted in accordance with standard procedures [35].

### Purification of mAbs

Antibodies were purified from culture fluid supernatants by use of HiTrap protein G HP columns (GE Healthcare). Columns were equilibrated with 0.1 M Tris-HCl, pH 8.0. Culture fluid supernatants were adjusted to pH 8.0 by the addition of 1 M Tris-HCl, filtered using a 0.45 µm membrane and loaded onto the column. After washing, using 0.1 M Tris-HCl, pH 8.0, column-bound mAbs were eluted using 0.1 M glycine, pH 2.5. Immediately, the eluted mAb was restored to pH 8.0 by the addition of 1 M Tris-HCl and dialysed into PBS buffer, pH 7.4. Protein concentration was estimated by measuring absorbance at 280/320 nm using a DU-650 spectrophotometer (Beckman). The mAb concentration was calculated on the basis of an absorbance of 1 Au_280_ corresponding to 0.75 mg/ml. This correlates to an estimated extinction coefficient of ∼200,000 M^−1^.

### Epitope Mapping Using Pepscan Analysis

Solid phase synthesis of peptides on a plastic surface and immunoscreening by ELISA was carried out at the laboratory of Pepscan Systems BV (Lelystad, The Netherlands) according to established procedures [37], [38]. In brief, complete sets of overlapping 15-mer peptides were synthesized covering the amino acid sequences of both ovine and bovine PrP. Each unique 15-mer peptide was incubated with purified antibodies (tested at a range of concentrations and optimized for each antibody tested) in an ELISA-formatted assay. Peptide sequences were considered antigenic when the absorbance values of two or more consecutive peptides were at least three times greater than that of the background. Background levels were calculated as the mean absorbance measured for 20 consecutive, low reactive peptides (whereby, the standard deviation was less than 20% of the mean absorbance).

### SDS-PAGE and Western Blotting

#### Recombinant protein

RecPrPs, typically 25–50 ng, were diluted in LDS sample buffer (Invitrogen) under reducing conditions and resolved using 12% NuPAGE Bis-Tris gels (Invitrogen) prior to transfer to PVDF membrane (Millipore) for 1 h 30 min at 1.2 cm^2^/A. Proteins tested were: recOvPrP ARQ allele (25–233 and 94–233), mouse (*Prnp* ^a^ allele, residues 23–230 murine sequence); hamster (wild type allele, residues 23–231); bovine (residues 25–241 bovine sequence) and human (M129, residues 23–230 human sequence).

#### Brain tissue and preparation of PK-resistant PrP^Sc^

Brain tissue from TSE-infected animals were homogenised in sterile PBS buffer, pH 7.2 producing a 10% (w/v) homogenate. Typically, 500 µl of homogenate was treated with sodium phosphotungstic acid (NaPTA) following digetsion with PK treatment as described in McCutcheon *et al.* [36] with the following deviations: brain samples were not given a clearing spin (80 g 1 min [39]) prior to the MgCl_2_ buffer and benzonase treatment and the final pellets were resuspended in 100 µl volumes. Proteins were separated using either 10 or 12% NuPAGE Bis-Tris gels (Invitrogen) and then transferred to PVDF membrane (Millipore) for 1 h 30 min at 1.2 cm^2^/A. A range of different species/TSE disease combinations were tested and are shown in Table S2, including ovine scrapie (naturally infected cases), ovine CH1641 scrapie, ovine atypical scrapie, ovine BSE, caprine scrapie and BSE, bovine BSE and deer BSE. All of these materials were provided by co-authors involved in the study with the exception of the scrapie-infected goat brain; bovine BSE macerate and BSE-infected goat brain (see acknowledgements).

#### Immunodetection of recPrP and PK-resistant PrP^Sc^ on PVDF membranes

Membranes were blocked using 5% (w/v) Marvel (non-fat) milk powder/PBS buffer, pH 7.2, containing 0.1% (v/v) (PBS-T) and then incubated overnight at 4°C with the different mAbs (typically at 1–0.1 µg/ml). After washing with PBS-T, the membrane was incubated in peroxidase-conjugated goat anti-mouse IgG (Sigma) for 1 h at room temperature (working dilution of 1∶20,000). After a final wash step, bound antibodies were visualised on ECL Hyperfilm (Amersham) using West Pico enhanced chemiluminescence substrate (Pierce). Hyperfilm was scanned and the digital image processed using Adobe Photoshop. Image processing was restricted to cropping, montage creation and annotation. Sha 31 [40], P4 [22] 6H4 ([24]) and 12B2 ([41]) were purchased from Bio-Rad/SPI-Bio, R-Pharma, Prionics and CVI, Wageningen UR respectively.

#### Brain tissue preparation and deglycosylation of PrP^C^ using peptide N–glycoside F (PNGase F)

Brain material for this work was obtained from 7 sheep in the Roslin Institute Cheviot flock [9]. Sheep had either ARQ/AHQ, ARQ/ARQ or AHQ/AHQ PRNP genotypes. PrP^C^ was extracted from brain (cortex) as described in [42]. In brief, tissue was manually homogenised in lysis buffer (containing 5% NP-40 (v/v), 12.1 mM sodium deoxycholate in PBS and protease inhibitors) to a 10% (w/v) homogenate. The homogenate was clarified by centrifugation and frozen until required. BCA (bicinchoninic acid) assays (Pierce) were performed on homogenates to establish total protein concentration. All reagents were provided with the kit and protocol followed according to the manufacturers instructions. PrP^C^ in brain was deglycosylated, resolved using SDS and immunoblotted as described in [42]. ROS-BC6 was used at a final concentration of 0.3 µg/ml in 0.5% blocking reagent and incubated overnight. Murine α-tubulin (NeoMarkers, Fisher) was used at a final concentration of 0.01 µg/ml in 0.5% blocking reagent and incubated for 1 hour.

### Immunohistochemistry

#### Immunohistochemistry (IHC) for detection of PrP^d^ in tissues from multiple host species with five different ROS- antibodies

Brain and lymphorecticular tissues from sheep, cattle, goats and deer naturally exposed or experimentally infected with different sources of scrapie or BSE, as described in detail in Table S2, were examined for the presence of PrP^d^ by IHC. The detailed IHC protocol has been described elsewhere and sections counterstained with Mayer’s haematoxylin [43], [44] and details on the final concentration of the five antibodies used is given in Table S3. Positive and negative control tissues were included to verify sensitivity and specificity of the IHC procedure.

#### Immunodetection of PrP^d^ in Scrapie Infected Mouse Brain using ROS-BH1

A modified version of methods described in [43] was used for IHC of murine tissues. Paraffin-embedded sections (7 µm) were cut from the brains of end-stage CV (C57Bl x VM F1) and conventional VM/Dk mice inoculated with 20 µl of 1% (w/v) ME7 or 87V-infected brain homogenate. Antigens were retrieved by autoclaving in a 0.1 M citric acid buffer (pH 6.4) for 15 min at 121°C before subsequently exposing to formic acid for 10 min. Sections were washed and endogenous peroxidase was blocked with 1% (v/v) H_2_O_2_ in methanol for 20 min. After washing, non-specific immunolabelling was blocked by incubation with an appropriate serum (10% v/v) for 20 min, before incubation overnight with mAbs ROS-BH1, 6H4 (Prionics, Lot H1001419M45) or TNP (IgG isotype control, BD Biosciences) antibodies at final concentrations of 0.2, 3 and 0.2 µg/ml respectively. Sections were subsequently incubated at room temperature for 1 h using an appropriate biotinylated secondary antibody and 30 min with ABC reagent (Vector Laboratories). Reactivity to the secondary antibody was visualised using diaminobenzidine (DAB, Vector Laboratories). Positive and negative tissue controls were included to verify sensitivity and specificity of the IHC procedure.

## Results and Discussion

### Antibody Generation and Isotyping

A panel of new anti-PrP antibodies was generated by immunising nine PRNP^−/−^ mice with an N-terminally truncated form of ovine recombinant protein (recOvPrP, residues 94 to 233, ARQ allele). Different immunisation strategies and boosting regimes were employed to attempt to generate antibodies with different properties (Table S1). Mice were immunised with recOvPrP, 94–233 combined with either Titre max, Quil A or Alum as adjuvant and boosts were performed every 4, 6 or 8 weeks after the initial priming immunisation i.e. mouse ID 0, in experimental group 1 was boosted every 4 weeks after the first immunisation. When antibody titres in tail blood reached maximal levels, spleens were harvested and resultant cell suspensions fused with a myeloma cell line. In the interests of time, spleens from mice with the identifications 1(1), 2(0) and 3(2) were pooled and a single fusion prepared (F782). Seven fusions were prepared in total. Each fusion produced 1000 viable clones of which the specificities for recOvPrP, PrP^C^ and PK-resistant PrP^Sc^ were tested. Four monoclonal antibodies were produced from each fusion and isotyping showed that all antibodies generated were IgG with variability only in the subclasses (IgG, IgG2a and IgG2b, Table 1). Of the 28 monoclonal antibodies generated, only 18 (listed in Table 1) were selected for further characterisation using a range of immunoassays, including immunohistochemistry, Western blotting and epitope mapping using Pepscan analysis.

**Table 1:** Identification of antibody isotypes, epitopes and cross reactivity to mammalian recombinant PrP.

Antibody	Isotype	Epitope	Group	Recombinant PrP					
				Ovine	Ovine	Murine	Hamster	Bovine	Human
				(25–233)	(94–233)	(23–230)	(23–231)	(25–241)	(23–230)
AE11	IgG1	^140^ **PLIHFG** ^145^	1	+	+	+	+	+	+
CF5	IgG2a	^140^ **PLIHFG** ^145^	1	+	+	+	+	+	+
IH9	IgG2a	^140^ **PLIHFG** ^145^	1	+	+	+	+	+	+
HC7	IgG1	^140^**PLIHFG** ^145^	1	NT	NT	NT	NT	NT	NT
EG6	IgG1	^147^DYE**DRYY**R^154^	2	+	+	+	+	+	–
DE3	IgG1	^146^NDYE**DRYY** ^153^	2	+	+	+	+	+	+
HC2	IgG1	^150^ **DRYY**RENM^157^	2	+	+	+	–	–	–
BC6	IgG1	^144^FGNDYE**DRYY**R^154^	2	+	+	+	+	+	–
BH1	IgG1	^143^HFGNDYE**DRYY**R^154^	2	+	+	+	+	+	+
FH10	IgG2a	^202^TETDIKIME^210^	3	+	+	+	+	+	+
JB10	IgG1	^222^(Q)RE**SQAYY(Q)** ^230^	4	+	+	–	–	+	–
DC12	IgG1	^224^E**SQAYYQ**R^231^	4	+	+	–	–	+	–
FH6	IgG1	^225^ **SQAYYQ** ^230^	4	+	+	–	–	+	+
JC4	IgG1	Unknown (ND)	–	+	+	+	+	+	+
EA6	IgG1	Unknown (ND)	–	+	+	–	–	+	–
FD12	IgG1	Unknown (ND)	–	+	+	+	–	–	–
EC9	IgG2a	Unknown (NT)	–	+	+	+	+	+	+
IH11	IgG2b	Unknown (NT)	–	+	+	+	+	+	+
Sha 31	IgG1	^148^YEDRYYRE^155^	NA	NT	NT	NT	NT	NT	NT
P4	IgG1	^93^WGQGGSH^99^	NA	NT	NT	NT	NT	NT	NT
6H4	IgG1	^147^DYEDRYYRE^155^	NA	NT	NT	NT	NT	NT	NT
12B2		^93^WGQGG^97^	NA	NT	NT	NT	NT	NT	NT

Antibodies which were shown to have similar binding motifs were assigned to distinct groups (1 to 4, whereby the common amino acid sequences are highlighted in bold/underlined text). For comparison, the epitopes and source of commercially available PrP-antibodies (Sha 31, P4 and 6H4) are indicated. The amino acid sequence of the core binding region of each antibody is indicted. The epitopes for ROS-JC4, ROS-EA6 and ROS-FD12 could not be determined (ND) using Pepscan, whereas ROS-EC9 and ROS-IH11 were not tested (NT) in pepscan analysis. Several mammalian species of recombinant PrP (typically 25 ng protein) were resolved using SDS-PAGE and probed with the mAbs post Western transfer**.** ‘+’ indicated antibodies which cross react and ‘–’ antibodies that did not cross react. ROS-HC7, Sha 31, P4, 6H4 and 12B2 were not tested (NT) in this assay.

### Antibody Binding Regions are within the Central Region and C-terminus of PrP

**Figure 1:**
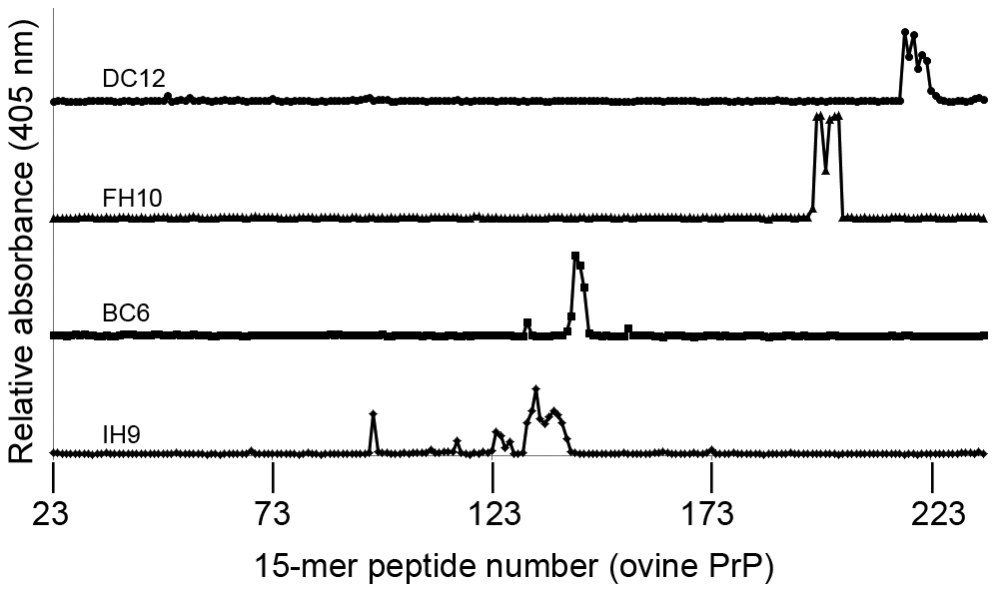
Four distinct binding regions are identified using Pepscan analysis. Antibodies were tested using solid-phase, PrP-based peptide Pepscan analysis. Y-axis represents absorbance; x-axis corresponds to the peptide set. Each peptide set is composed of 15 amino acids, whereby set number 1 starts with the residue 1 in PrP; peptide set 2 starts at residue 2 in PrP and so on. Positive reactions between antibodies and surface-bound peptides resulted in an increase in absorbance (405 nm) which could be discriminated from background/‘noise’ readouts. Here we show increased absorbance indicative of the core binding regions from individual representatives of the four distinct antibody groups identified.

To map the epitopes of the antibodies, Pepscan analysis was undertaken by use of overlapping, 15-mer peptides based on the ovine sequence of PrP. Figure 1 shows representative outputs of Pepscan analysis for the antibodies ROS-DC12, ROS-FH10, ROS-BC6 and ROS-IH9; increases in the relative absorbance above a pre-defined threshold indicate antibody binding a particular series of overlapping peptides. For example, ROS-BC6 bound to peptides 141 to 144, allowing the epitope to be localised within the sequence ^144^FGNDYEDRYYR^154^. The core epitopes of antibodies which were reactive in Pepscan are summarised in Table 1, alongside the commercially available antibodies Sha 31, P4, 6H4 and 12B2.

By comparing the epitope of all the antibodies mapped, it became clear that they segregate into four distinct binding regions, designated as groups 1–4. All group 1 mAbs bind to the amino acid sequence ^140^PLIHFG^145^, which precedes helix 1 of the PrP molecule. Group 2 antibodies recognise an area spanning amino acids 143–157, within helix 1, and in which the DRYY motif formed the consensus binding area for those antibodies. The epitope of ROS-FH10 was identified at the start of helix 3, proximal to the second glycosylation site, and was composed of amino acids 202–210, TETDIKIME. The epitope of the group 4 antibodies was mapped to a sequence in the extreme C-terminal region of PrP, spanning residues 222–231, RESQAYYQR, and in which the common consensus sequence was ^225^SQAYYQ^230^. The positioning of these four distinct core binding regions on the tertiary structure of the globular domain for ovine PrP is illustrated in Figure S2. Not all of the antibodies tested using Pepscan mapped to linear binding sequences (i.e. ROS-FD12, ROS-EA6 and ROS-JC4), implying that these antibodies may recognise conformational epitopes composed of amino acids that are far apart in the linear sequence but spatially proximal in folded PrP. Additionally, not all antibodies were tested in Pepscan analysis (ROS-IH11 and ROS-EC9).

### Multi-species Reactivity against Bacterially-expressed, Mammalian PrP

**Figure 2:**
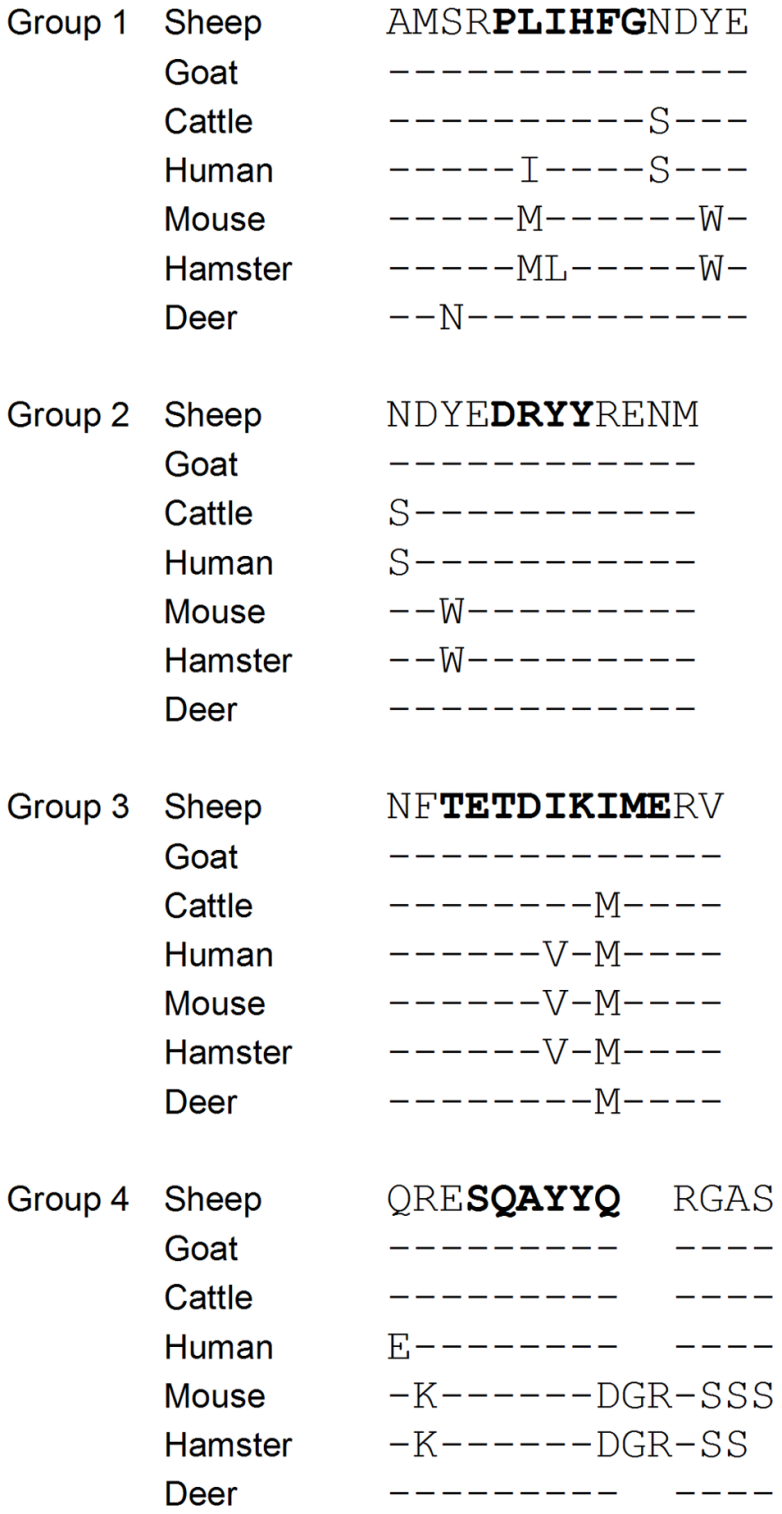
Partial alignment of PrP sequences studied in this work. Reference sequences were obtained from UniProt and aligned using ClustalX using the sheep sequence as the reference. Segments of the sequence corresponding to the core epitopes for the 4 groups of antibodies are shown in bold with additional flanking sequence for reference. Where amino acids are the same as sheep this is indicated with a dash; where amino acids differ these are explicitly stated, or a gap is left where no amino acids align.

**Figure 3:**
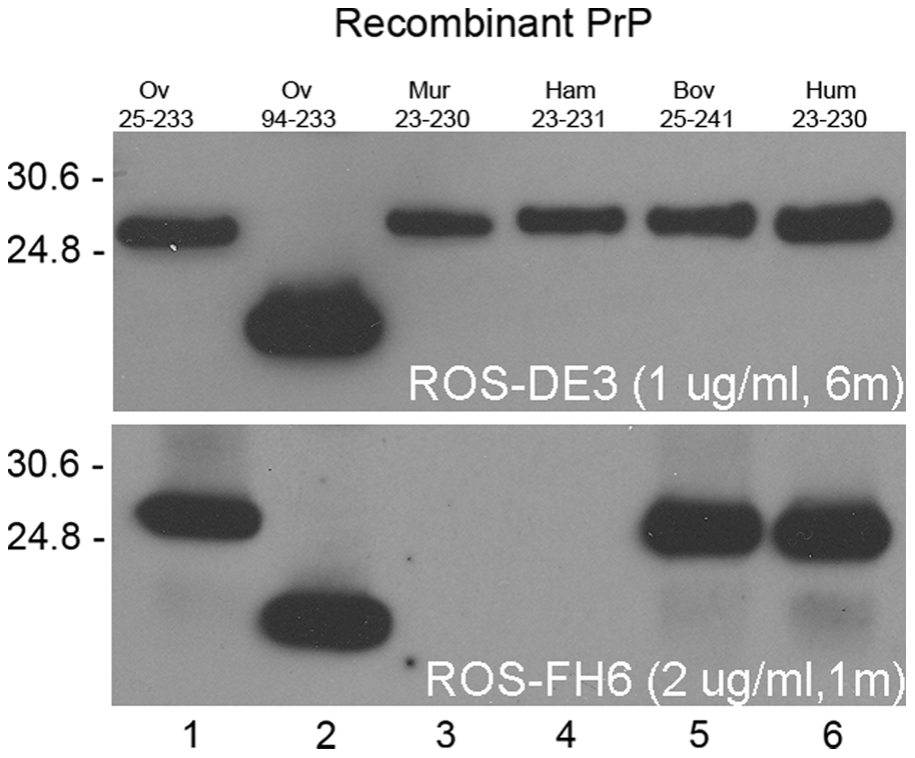
Binding of ROS-FH6 (compared to ROS-DE3) to mammalian PrP is affected by the sequence. Immunoblots of recOvPrP residues 25–233 ovine sequence (lane 1), recOvPrP residues 94–233 ovine sequence (lane 2), murine (Mur) PrP residues 23–230 murine sequence (lane 3), hamster (Ham) residues 23–231 hamster sequence (lane 4), bovine (Bov) PrP residues 25–241 bovine sequence (lane 5) and human (Hum) PrP residues 23–230 human sequence(lane 6) when probed with ROS-DE3 and ROS-FH6. ROS-DE3 cross reacts with all species of mammalian PrP tested, whereas ROS-FH6 failed to bind to murine and hamster PrP. Molecular weight markers are shown in kDa. Exposure time is shown in minutes (m).

Recombinant PrP corresponding to sheep (25–933 and 94–233 variants, 25 kDa and 17 kDa respectively) murine, hamster, bovine and human PrP sequences were resolved using SDS-PAGE and immunoblotted with the antibodies. Many antibodies cross-react with all recPrPs tested, probably as a result of the high degree of homology of the amino acid sequence of each protein in the regions of the antibody epitopes (Figure 2). A representative example of the Western blot assay is shown in Figure 3 using ROS-DE3 and ROS-FH6 and a summary of the species cross reactivity derived from these studies is detailed in Table 1. However, some antibodies showed clear differential binding to the recombinant protein panel in the Western blot assay. For example, ROS-JB10, ROS-DC12, ROS-FH6 and ROS-EA6 bound to ovine and bovine recPrP but not to murine or hamster. Whilst the epitope for ROS-EA6 was not identified by use of Pepscan analysis, the binding regions for ROS-JB10, ROS-FH6 and ROS-DC12 were mapped to a common motif ^225^SQAYYQ^230^. It is notable that in the hamster and murine sequences, aspartic acid replaces glutamine at residue 230, implying that residue 230 is critical for antibody binding in this region. For these antibodies, it was also interesting to note the differential reactivity to human PrP by ROS-FH6 compared to both ROS-JB10 and ROS-DC12. The reason for this is not clear, but is conceivable that the mapping studies, in general, may not be wholly definitive and that the ‘complete’ epitope for ROS-JB10, ROS-DC12 and ROS-FH6 may include a wider region than that defined by the Pepscan analysis. In this context, the data could be reconciled in terms of different amino acids expressed at residues 222 (Q/E) and/or 223 (R/K) in ovine and human sequences. ROS-HC2, which mapped to the ovine sequence ^150^DRYYRENM^157^, bound to ovine and murine recombinant proteins only. The failure to react with the bovine, murine and hamster proteins appears to be associated with amino acid differences at residues 151 (Y/W) and potentially at 149 (N/S). ROS-FD12 also showed a similar pattern of species specificity to ROS-HC2, though the epitope for ROS-FD12 was not determined using Pepscan.

### Arginine at Codon 154 is Critical for Antibody Binding of ROS-BC6

**Figure 4:**
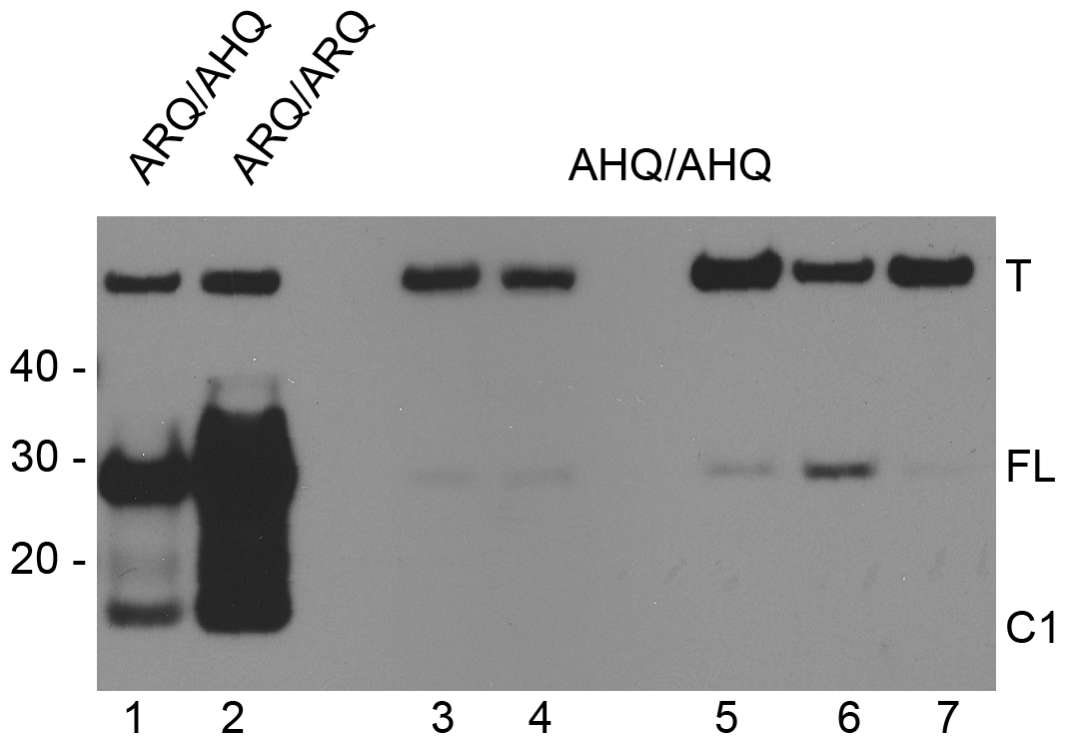
ROS-BC6 requires arginine (R) at codon 154 for binding to C1 PrP^C^. Representative image of mature-length (FL) and C1 PrP^C^ (C1, deglycosylated forms) extracted from brain of seven sheep of either ARQ/AHQ (lane 1), ARQ/ARQ (lane 2) or AHQ/AHQ (lanes 3–7) PRNP genotypes. Membrane was probed with ROS-BC6 (0.3 µg/ml) simultaneously with anti-tubulin (T, 0.01 µg/ml). ROS-BC6 binds specifically to mature-length and C1 PrP in brain from ARQ/AHQ and ARQ/ARQ sheep but not from AHQ/AHQ sheep. Molecular weight markers are shown in kDa, exposure time was 10 minutes.

The epitope for ROS-BC6 was mapped to amino acids ^144^FGNDYEDRYYR^154^ (Table 1) and this antibody is used extensively in our laboratory for routine analysis of PrP^C^ and PrP^Sc^ in tissues collected from sheep infected with scrapie and/or BSE as well as healthy controls. It is known that ovine PrP^C^ can undergo proteolytic cleavage [45], resulting in the generation of a C-terminal fragment designated as C1. We have used ROS-BC6 to show that the levels of C1-PrP (as a percentage of total PrP) varied in accordance with *PRNP* genotype, whereby the highest levels of C1 were seen in ARR/ARR sheep compared to both VRQ and ARQ homozygotes. [42]. To further that analysis, we recently investigated the relative amounts of C1-PrP^C^ in sheep of ARQ/AHQ, ARQ/ARQ and AHQ/AHQ genotypes. Figure 4 shows a representative Western blot probed with ROS-BC6 (0.3 µg/ml) and tubulin (T, 0.01 µg/ml) antibodies simultaneously according to the published methods [42]. A marked reduction in binding of ROS-BC6 to full-length PrP (∼28 kDa) in brain from AHQ/AHQ sheep compared to both ARQ/ARQ and ARQ/AHQ was seen (comparison of lanes 3–7 with lanes 2 and 1 respectively). No C1-PrP (∼17 kda) was detected in brain from any of the AHQ/AHQ sheep tested, even upon longer exposure times. Furthermore, coupling the data from this experiment and published findings with epitope mapping result for ROS-BC6 identified that arginine at codon 154 as a critical residue for reactivity in ovine PrP.

### PK-resistant PrP^Sc^ from Multiple TSE Agents is Detected Using the ROS Antibodies: Comparative Analysis with Commercially Available Antibodies

One of the main aims of the research we undertake relates to the detection of PK-resistant PrP^Sc^ extracted from tissues from animals infected by both experimental and natural routes. Therefore, finding optimal reagents that give superior data in aiding that objective is critical. To this end, we selected a sub panel of antibodies to test their ability to detect PK-resistant PrP^Sc^/PrP^d^ (defined as disease-associated PrP in immunohistochemistry assays given that tissue sections are not treated with PK) from animals affected with different TSE agents (Table S2) in Western blotting and immunohistochemistry assays respectively.

**Figure 5:**
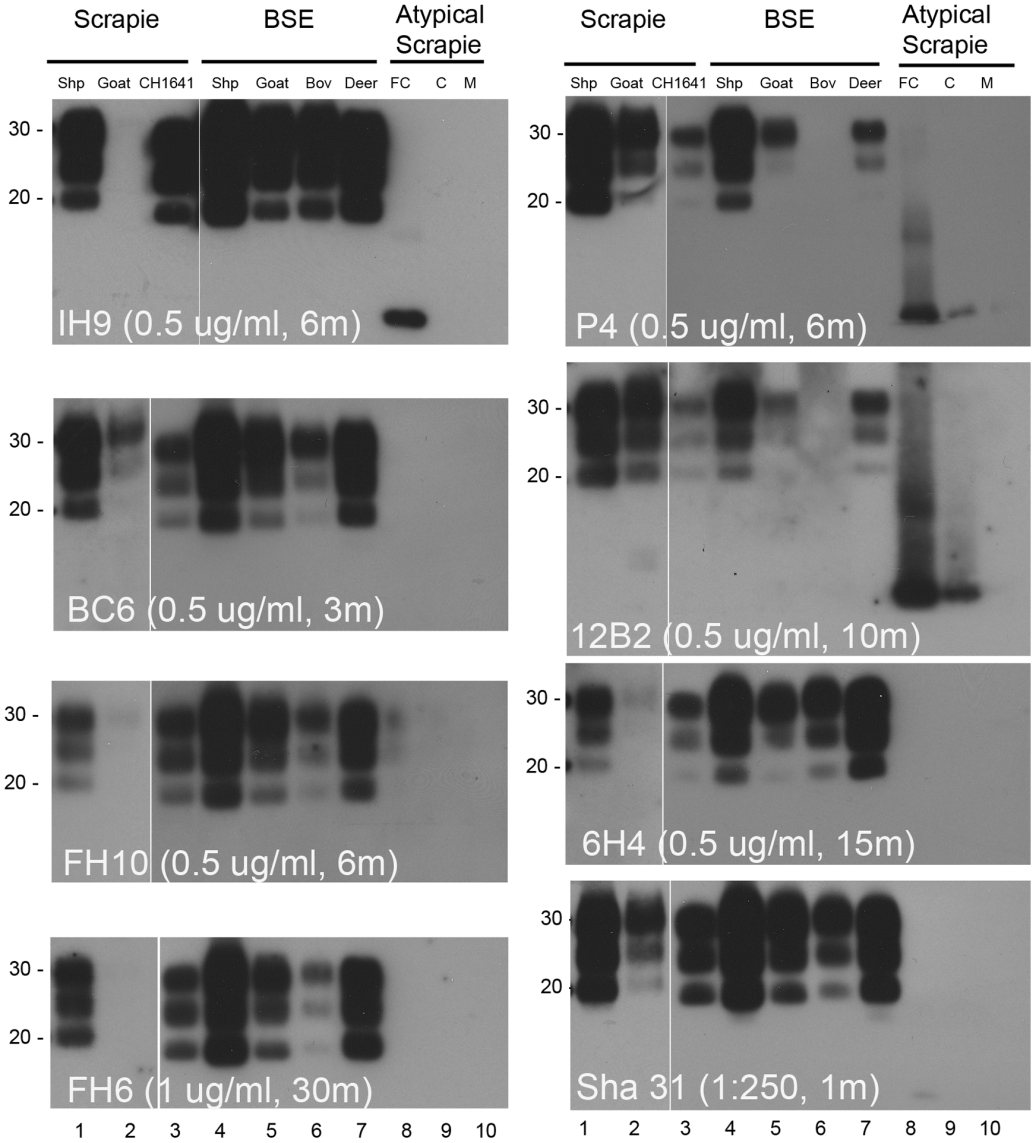
Testing the mAb specificity for different TSE agents. PrP^Sc^ was extracted from the brain of a range of host animals infected with different TSE agents, treated with PK and detected using SDS-PAGE and immunoblotting with the new mAbs and selected commercially available antibodies. Natural sheep (Shp) scrapie (lane 1), goat scrapie (lane 2), CH1641 scrapie (lane 3), sheep (Shp) BSE (lane 4), goat BSE (lane 5), cow (Bov) BSE (lane 6), deer BSE (lane 7) and atypical scrapie - frontal cortex (FC), cerebellum (C) and medulla (M) (lanes 8, 9 and 10 respectively). Molecular weight masses are shown in kDa. Each immunoblot shows antibody concentration (µg/ml) and exposure time to film (minutes, m).

Figure 5 shows a series of representative immunoblots obtained when PK-treated samples from TSE-infected animals were probed with antibodies ROS-IH9, ROS-BC6, ROS-FH10 and ROS-FH6, compared to the commercially available antibodies 6H4, Sha 31, 12B2 and P4. Where possible, we standardised the concentrations of antibodies used to a final concentration of 0.5 µg/ml. The 3 antibody-reactive protein bands correlating to the di-, mono- and un-glycosylated forms of PrP resolved between 20–30 kDa. A lower molecular weight band, characteristic of atypical scrapie, resolved ∼10 kDa. The 10 kDa band has been shown, for Nor 98 strain of atypical scrapie and using a monoclonal antibody-based epitope mapping, to contain amino acids spanning residues 93–153 of ovine PrP [6] and was detected by commercially-available mAbs P4 and 12B2. It could have been expected that antibodies containing epitopes assigned to group 1 and 2 (residues 140–145 and147–154 respectively) would be able to detect the 10 kDa band characteristic of atypical scrapie in sheep. However, only ROS-IH9 was capable of binding to this band (frontal cortex, lane 8 in the IH9 panel). This result was confirmed by testing frontal cortex, cerebellum and medulla samples from greater numbers of sheep with atypical scrapie with ROS-IH9 and ROS-BC6 (Tan *et al.,* in preparation). Of note, the genotype of the sheep with atypical scrapie was homozygous for phenylalanine at codon 141, whilst the ovine sequence used for epitope mapping contained leucine at the equivalent position, indicating that ROS-IH9 binds the 10 kDa band when either amino acid is present.

All of the ROS antibodies cross reacted with the majority of strains/species combinations tested. The notable exception observed was reactivity to PrP^Sc^ from goat scrapie (lane 2, all panels). The reason for this is not clear, though we suggest that this particular goat sample has lower levels of PK-resistant PrP^Sc^ than other goat samples examined (not shown) and to the other samples tested in this assay. Equally, these data show that the antibodies do not have an equal affinity for all TSE agents; only antibodies with the highest affinity will bind i.e. ROS-BC6, ROS-IH9, 12B2, Sha 31 and P4. We show that the reactivity profile of the ROS antibodies (used singly or in combination) tested in this assay is comparable to the profiles obtained using either of the commercially-available antibodies. Of more significance than the broad reactivity of these antibodies to different TSE agents and in a range of infected host animals, is the comparable and in some cases improved sensitivity of binding compared to the commercially available mAbs. For example, ROS-IH9 appeared to react with the same specificity to the TSE agents as 6H4/12B2/P4 but with improved sensitivity i.e. better reaction to BSE samples (lanes 4 to 7) compared to 12B2 and P4 (assessed by a combination of the concentration of antibody used and the time of exposure required to give the same reaction profile). That said ROS-IH9 showed a similar reactivity profile compared to Sha 31 for the agents tested. The exceptions noted were the positive reaction of ROS-IH9 for atypical scrapie and the positive reaction of Sha 31 for goat scrapie sample.

### PrP^d^ Detection by IHC in a Range of Ruminant Hosts Infected with Classical or Atypical Scrapie or BSE

Antibodies ROS-IH9, ROS-BH1, ROS-BC6, ROS-FH6 and ROS-FH10 were also tested in IHC examinations of paraffin sections from both central nervous system (CNS) and lymphorecticular (LRS) tissues from the animals listed in Table S2. We found that ROS-IH9 and ROS-BH1, used either singly or in combination, provided superior results compared to commercially available antibodies, in terms of sensitivity and ability to detect a range of different PrP^d^ types. Assays on central nervous system (CNS) tissues were independently scored from 0 (no labelling) to 3 (most intense labelling), and results are given in Table S3 for large animal infection models and Table S4 for small animal infection models. Overall, ROS-IH9 gave the highest combined score compared to the other antibodies tested. In the case of animals tested in these assays, ROS-IH9 was capable of detecting PrP^d^ in the CNS of all agent/host combinations, whereas the other antibodies failed to detect PrP^d^ in cases of atypical scrapie, CH1641 scrapie or both.

**Figure 6:**
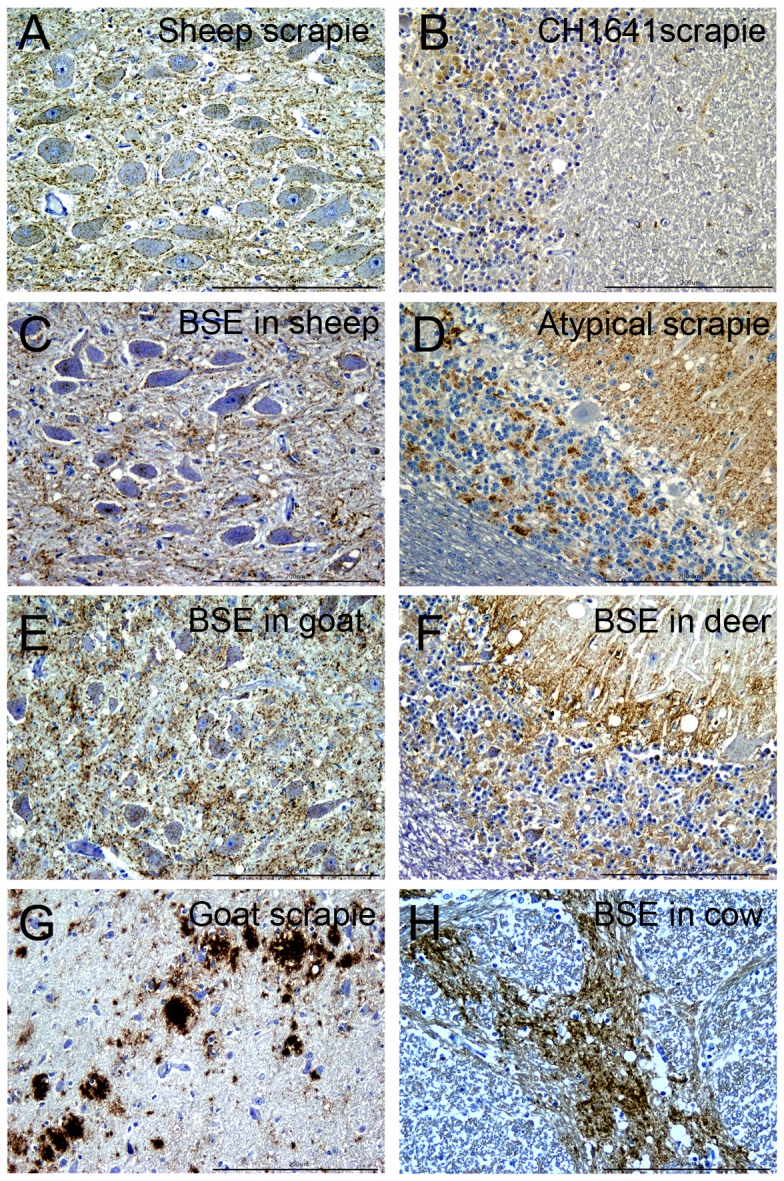
Different profiles of PrP^d^ staining are evident when brain sections from TSE-infected sheep, goat, cow and deer were stained with ROS-IH9. Labelling for PrP^d^ in the brain (indicated by brown staining) from several ruminant species infected with different TSE agents using the antibody ROS-IH9 (0.0625 µg/ml). Experimental classical scrapie in sheep specifically the dorsal motor nucleus of the vagus nerve (DMNV, panel A); CH1641 scrapie in sheep (cerebellum, panel B); cattle BSE in sheep (DMNV, panel C); atypical scrapie in sheep (cerebellum, panel D); goat BSE in goat (DMNV, panel E); cattle BSE in red deer (cerebellum, panel F); classical scrapie in goat (hypothalamus, panel G); natural BSE in cattle (spinal tract of the trigeminal nerve, panel H). Scale bar = 200 µm.

Figure 6 illustrates a range of PrP^d^ types in the CNS of several TSE examples in different species after labelling with ROS-IH9. A range of staining patterns [44] were observed within the dorsal motor nuclei of the vagus nerve in TSE-affected sheep. For example, for natural sheep classical scrapie, punctuate intraneuronal, perineuronal and widespread fine particulate neuropil labelling was observed in the dorsal motor nucleus of the vagus nerve (DMNV, Figure 6A). A similar PrP^d^ profile was seen in sheep and goats experimentally infected with BSE albeit with different intensities of labelling staining within neurones (Figure 6C and 6E respectively). For agents targeting the cerebellum, diffuse labelling of the granular cell layer as well as intracellular labelling of glial cells in the granular and molecular cells layers was seen in sheep infected with the CH1641 strain of scrapie (Figure 6B). For sheep with atypical scrapie, ROS-IH9 identified widespread accumulation of PrP^d^ throughout the granular, molecular and white matter of the cerebellum. In red deer infected with BSE, intracellular labelling of Purkinje cells and Golgi neurons together with intense extracellular deposits of PrP^d^ throughout the granular and molecular cell layers was observed. Figure 6G shows strongly-labelled, conspicuous PrP^d^ masses, coalescing and plaque-like, at the level of the hypothalamus of a goat with nature scrapie and Figure 6H shows coalescing PrP^d^ aggregates in the spinal tract of the trigeminal nerve of a case of cattle BSE. Comparative negative control tissue sections from sheep, goat, deer and cow stained with ROS-IH9 (at a final concentration of 0.063 µg/ml) are shown in Figure S3, panels A to D respectively.

### ROS-BH1 gives a more Sensitive Detection of Mouse Scrapie Agents Compared to 6H4

**Figure 7:**
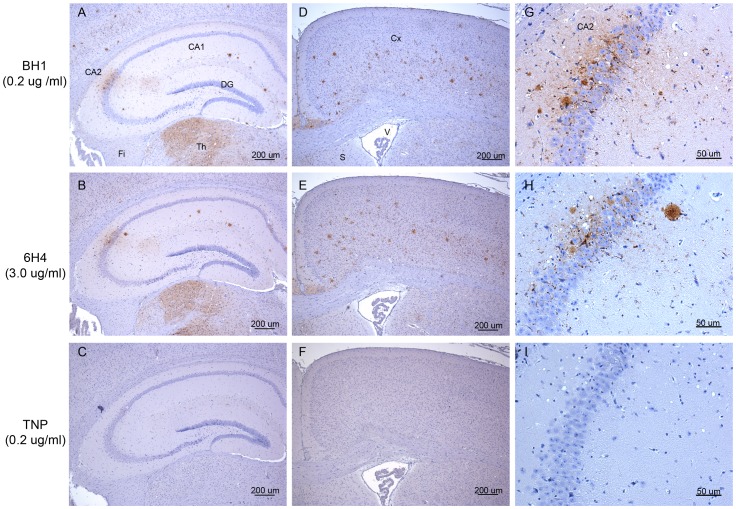
ROS-BH1 stains PrP^d^ deposited after 87V infection in mice in a more sensitive manner compared to 6H4. Staining of PrP^Sc^ during 87V (scrapie) infection in the cortical hippocampus, the cortex and the CA2 region of the hippocampus in mice using ROS-BH1 (0.2 µg/ml, panels A, D and G), 6H4 (3.0 µg/ml, panels B, E and F) and the IgG1 isotype control antibody, TNP (0.2 µg/ml, panels C, F and I). Panels, G H and I are higher magnification images of the CA2 region. Neuroanatomical landmarks are identified as follows: Fi – fimbria; Th - thalamus; DG - dentate gyrus; CA2 - CA2 region of the hippocampus; CA1 - CA1 region of the hippocampus; Cx - cortex; V - ventricle and S - septum. Scale bars are indicated in µm. ROS-BH1 gave more sensitive detection of PrP^d^ in this assay compared to 6H4 whilst retaining an identical specificity and profile of staining to that of 6H4.

Figure 7 shows representative PrP^d^ deposition in the hippocampus and cortex during 87V infection in mice, when sections were stained with either ROS-BH1 or 6H4. Slides were coded and independently assessed. Using ROS-BH1 fine, punctate PrP staining in association with plaques were evident in the cortex at the level of the caudate nucleus, the cortex at the level of the hippocampus and within the hippocampus. Fine punctate staining was evident in the thalamus and hypothalamus (panels A, D and G). Though not shown here, PrP staining was also observed within the colliculous (fine punctate and pericellular staining), brainstem (fine punctate) and the cerebellum (plaques within the granular cell layer). In relative terms, the intensity of staining observed between ROS-BH1 and 6H4 was similar, with higher scores recorded for ROS-BH1 within the caudate nuclei, hypothalamus and colliculous compared to 6H4 (Table S4). Importantly, the same profile and pattern of PrP staining was observed when we used ROS-BH1 as that obtained when serial sections were stained with 6H4 (comparison of panels A, D and G to B, E and H respectively), which we have routinely used for such analysis. However we used ROS-BH1 at a 15 fold lower concentration than 6H4, indicative of the level of sensitivity that can be achieved with this antibody. No staining was observed when serial sections were stained with TNP, the isotype control for both 6H4 and ROS-BH1 (panels, C, F and I). To confirm the results with ROS-BH1 were not co-incidental or restricted to a single TSE agent, we repeated this assay using tissue from mice infected with ME7 scrapie (Figure S1 and Table S4). The same outcome in terms of improved sensitivity of detection of PrP^d^ by ROS-BH1, compared to 6H4, were obtained.

## Conclusions

This paper details the production, extensive characterisation and applications of a panel of anti-prion protein antibodies (designated as the ‘ROS’-series of antibodies). Notably, when tested alongside the current world-leading antibodies, several of these new antibodies (used singly or in combination) provide comparable or improved results in immunoassays commonly used for diagnosing TSE infection in multiple host species and for investigating the biological processes underlying these diseases. This is highly significant in terms of the cost of running high-throughput screening assays or routine assays spanning long experiment times typically associated with TSE infection in animal models.

These antibodies have been used extensively in our laboratories and distributed to other research labs for collaborative use, resulting in several primary publications relating to: the identification of a new form of prion protein in BSE-affected cattle (using ROS-JB10 [46]), the production of a PrP fragment 114–188 from PrP^Sc^ in heterozygous sheep (using ROS-FH6 [47]), assessment of relative C1 PrP^C^ levels in sheep of different PrP genotypes (using ROS-BC6, ROS-FH10 and ROS-JB10 [42]), the identification of infection and distribution of PrP^Sc^ in tissues of sheep experimentally infected with BSE blood components (using ROS-BC6 [48]), the effect of *PRNP* genotype on the incubation times in sheep with BSE (using ROS-BC6 [49]) and the characterisation of PrP deposition and pathology associated with BSE infection in aged and young mice (using ROS-BH1) [50].

Collectively, epitopes covered by the ROS-antibodies bind to discrete regions within PrP, spanning residues 140–154, 202–210 and 222–230. This is of both functional and biological significance, since data emanating from the use of antibodies with different epitopes within PrP have yielded significant advancements in prion biology relating to: diagnostic regimes for animal TSE disease (using 6H4 [22], [24], [51], [52] or human TSEs (using KG9 singly or in combination with other antibodies i.e. 3F4, ICMS35, BG4 [53]–[56]); immunological characterisation of pathology associated with established and emerging TSEs, such as immunophenotyping of atypical scrapie using an array of antibodies [2] or identification of the shared characteristic of Nor98 scrapie and human Gerstmann-Straussler-Scheinker disease (GSS) [6]; the use of sequences in the prion protein which are species specific (using 3F4 or antisera raised against synthetic peptides [14], [25], [57]); the discrimination of TSE strains in large and small animal models, for example natural scrapie, BSE and CH1641 scrapie (using P4 and 66.94ba [58], 6H4/P4 [59], P4 [60]), using triplex immunostaining with L42, 12B2 and SAF84 [61] and using multiple antibody panels [5], [41], [62]–[67]; the characterisation and classification of human prion disease [68]–[71]; the characterisation of structural elements within PrP, such as deciphering accessibility and exposure of epitopes following the conversion of PrP^C^ to PrP^Sc^ or those that are PrP^Sc^-specific i.e. the Tyr-Tyr-Arg motif [72], 15B3 [24], 6H10 [73]–[77] and identifying potential structural intermediates in the PrP^C to^ PrP^Sc^ transition [78]–[82].

Given the coverage of epitopes collectively represented by the ROS-antibodies, it is conceivable that novel C-terminal truncated forms of PrP may be identified by mapping studies using ROS-FH10, ROS-JB10, ROS-DC12 or ROS-FH6. Furthermore, since the primary reagents used in many current screening assays and diagnostic tests for prion diseases are still largely antibodies, a new reservoir of high-affinity reagents will help to improve the sensitivities of such approaches for both humans and animals. Knowledge of the epitopes represented by the ROS antibodies allows for multiple combinations of antibody pairs to be evaluated as capture and detector in sandwich-type immunoassays, with or without pre-treatments using the enzyme PK or used to identify the differences in exposure of cell-associated PrP epitopes in blood from healthy or TSE infected individuals [83]–[88] or for immune-capture of PrP^Sc^/misfolded forms of PrP present in blood [89].

Towards a therapeutic approach, some drug therapies, such as pentosan polysulphate and quinacrine, have shown promising outcomes in animal studies [90], [91], however they have been of limited success in a clinical setting, in part because of toxicity effects, inability to cross the blood-brain barrier or because benefits are only evident if treatment is initiated around the time of infection. A different strategy could be the use of monoclonal antibodies for the prevention or treatment of prion diseases, reviewed in [12], [92], [93] and described by Madampage *et al*. [94]. Favour has moved towards passive immunisation as an approach, to reduce/prevent prion protein replication and disease progression, as opposed to active immunisation due to the potential unwanted autoimmune effects. To be of significant benefit this strategy may require antibodies that specifically recognise abnormal forms of PrP, thereby reducing cross reactivity with abundant levels of cellular forms of the prion protein. In addition, it is important that anti-PrP antibodies used therapeutically do not induce apoptosis of neurones [95], [96] and the ROS series of antibodies are currently being trialled for their potential as therapeutic agents.

To achieve universal goals within the TSE field, such as diagnosis of established and emerging TSE agents and achieving new regimes for disease treatment and prevention, we continue to invite sharing of our new monoclonal antibodies through collaborative projects and/or independent use following purchase from the TSE resource centre (http://www.roslin.ed.ac.uk/tseresourcecentre) at The Roslin Institute.

## Supporting Information

Figure S1 ROS-BH1 stains PrP^d^ deposited after ME7 infection in mice in a more sensitive manner than 6H4. Staining of ME7 (scrapie) infection in the cortical hippocampus and the CA2 region of the hippocampus in mice using ROS-BH1 (0.2 µg/ml, panels A and D), 6H4 (3.0 µg/ml, panels B and E) and the IgG1 isotype control antibody, TNP (0.2 µg/ml, panels C and F). Neuroanatomical landmarks are identified as follows: Fi – fimbria; Th - thalamus; DG - dentate gyrus; CA2 - CA2 region of the hippocampus; CA1 - CA1 region of the hippocampus. Scale bars are indicated in µm. ROS-BH1 gave more sensitive detection of PrP^d^ in this assay compared to 6H4 whilst retaining an identical specificity and profile of staining to that of 6H4.
(DOCX)

Figure S2 Schematic ribbon diagram of the tertiary structure of the globular domain of PrP. The structure is based on that solved by crystallography by Haire et al [1] for ovine recombinant PrP and spans residues 128 to 233. Coordinates were obtained from the Protein Data Bank (code 1UW3) and were rendered using MolMol [2] and Povray. The common core binding regions for each group of antibodies are displayed as spheres representing the α-carbon atoms of the residues involved in the binding epitope.
(DOCX)

Figure S3 Comparative negative control tissue sections from sheep, goat, deer and cow stained with ROS-IH9. Panel A shows the dorsal motor nuclei of the vagus nerve (DMNV) at the level of the obex from sheep. Panel B shows the DMNV at the level of the obex from goat. Panel C shows cerebellum from deer. Panel D shows spinal tract from cow. Tissues were obtained from animals known not to be infected with a TSE. All tissues sections were stained with ROS-IH9 at a final concentration of 0.063 µg/ml. No PrP^d^ labelling (as indicated by the absence of brown staining) was observed in the tissue sections tested. Scale bars = 200 µm.
(DOCX)

Table S1 Immunisation strategy.
(DOCX)

Table S2 Details of ruminant species used to assess five ROS- antibodies by Western blotting (WB) and immunohistochemistry (IHC).
(DOCX)

Table S3 Comparative analysis of subjective scoring for PrP^d^ using five different ROS- antibodies in TSE-affected ruminants.
(DOCX)

Table S4 Arbitrary scoring of PrP^d^ in ME7 and 87V scrapie, using ROS-BH1 and 6H4.
(DOCX)
