# Supplementary material for: Prion Protein-Specific Antibodies that Detect Multiple TSE Agents with High Sensitivity
Source: PLoS One. 2014 Mar 7;9(3):e91143. doi: 10.1371/journal.pone.0091143 (PMC3946747; doi:10.1371/journal.pone.0091143)

**Figure S1: ROS-BH1 stains PrP^d^ deposited after ME7 infection in mice in a more sensitive manner than 6H4**

Staining of ME7 (scrapie) infection in the cortical hippocampus and the CA2 region of the hippocampus in mice using ROS-BH1 (0.2 µg/ml, panels A and D), 6H4 (3.0 µg/ml, panels B and E) and the IgG1 isotype control antibody, TNP (0.2 µg/ml, panels C and F). Neuroanatomical landmarks are identified as follows: Fi – fimbria; Th - thalamus; DG - dentate gyrus; CA2 - CA2 region of the hippocampus; CA1 - CA1 region of the hippocampus. Scale bars are indicated in µm. ROS-BH1 gave more sensitive detection of PrP^d^ in this assay compared to 6H4 whilst retaining an identical specificity and profile of staining to that of 6H4.


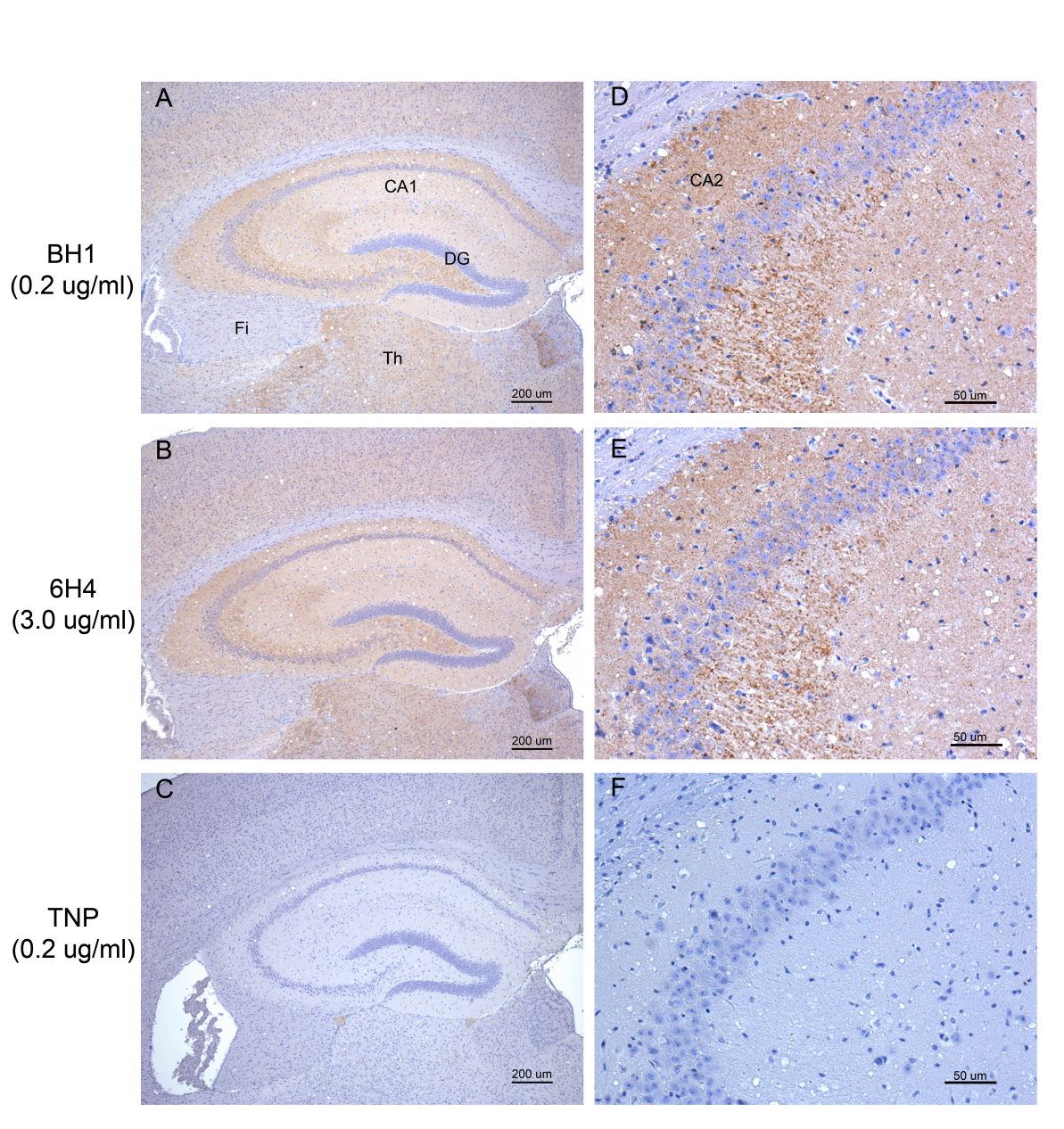

Supplement: Figure S1 — ROS-BH1 stains PrPd deposited after ME7 infection in mice in a more sensitive manner than 6H4. Staining of ME7 (scrapie) infection in the cortical hippocampus and the CA2 region of the hippocampus in mice using ROS-BH1 (0.2 µg/ml, panels A and D), 6H4 (3.0 µg/ml, panels B and E) and the IgG1 isotype control antibody, TNP (0.2 µg/ml, panels C and F). Neuroanatomical landmarks are identified as follows: Fi – fimbria; Th - thalamus; DG - dentate gyrus; CA2 - CA2 region of the hippocampus; CA1 - CA1 region of the hippocampus. Scale bars are indicated in µm. ROS-BH1 gave more sensitive detection of PrPd in this assay compared to 6H4 whilst retaining an identical specificity and profile of staining to that of 6H4. (DOCX) [file pone.0091143.s001.docx]
