# Supplementary material for: Prion Protein-Specific Antibodies that Detect Multiple TSE Agents with High Sensitivity
Source: PLoS One. 2014 Mar 7;9(3):e91143. doi: 10.1371/journal.pone.0091143 (PMC3946747; doi:10.1371/journal.pone.0091143)
Supplement: Figure S2 — Schematic ribbon diagram of the tertiary structure of the globular domain of PrP. The structure is based on that solved by crystallography by Haire et al [1] for ovine recombinant PrP and spans residues 128 to 233. Coordinates were obtained from the Protein Data Bank (code 1UW3) and were rendered using MolMol [2] and Povray. The common core binding regions for each group of antibodies are displayed as spheres representing the α-carbon atoms of the residues involved in the binding epitope. (DOCX) [file pone.0091143.s002.docx]

**Figure S2: Schematic ribbon diagram of the tertiary structure of the globular domain of PrP.** The structure is based on that solved by crystallography by Haire et al [[1](#_ENREF_1)] for ovine recombinant PrP and spans residues 128 to 233. Coordinates were obtained from the Protein Data Bank (code 1UW3) and were rendered using MolMol [[2](#_ENREF_2)] and Povray. The common core binding regions for each group of antibodies are displayed as spheres representing the α-carbon atoms of the residues involved in the binding epitope.


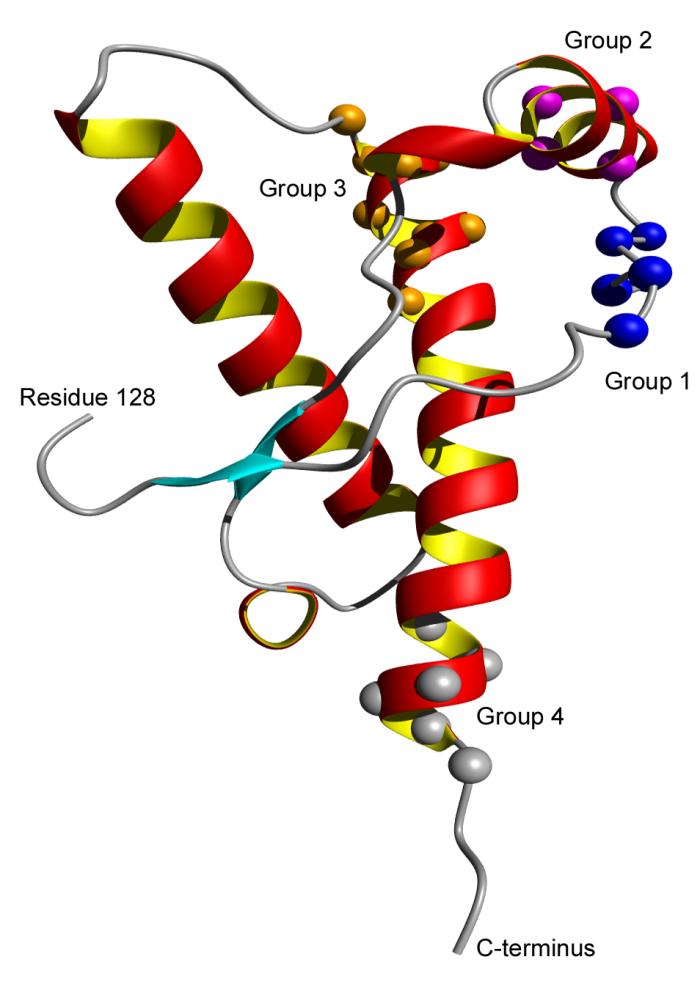


References

1. Haire LF, Whyte SM, Vasisht N, Gill AC, Verma C, et al. (2004) The crystal structure of the globular domain of sheep prion protein. J Mol Biol 336: 1175-1183.

2. Koradi R, Billeter M, Wuthrich K (1996) MOLMOL: a program for display and analysis of macromolecular structures. J Mol Graph 14: 51-55, 29-32.
