# Supplementary material for: Prion Protein-Specific Antibodies that Detect Multiple TSE Agents with High Sensitivity
Source: PLoS One. 2014 Mar 7;9(3):e91143. doi: 10.1371/journal.pone.0091143 (PMC3946747; doi:10.1371/journal.pone.0091143)
Supplement: Figure S3 — Comparative negative control tissue sections from sheep, goat, deer and cow stained with ROS-IH9. Panel A shows the dorsal motor nuclei of the vagus nerve (DMNV) at the level of the obex from sheep. Panel B shows the DMNV at the level of the obex from goat. Panel C shows cerebellum from deer. Panel D shows spinal tract from cow. Tissues were obtained from animals known not to be infected with a TSE. All tissues sections were stained with ROS-IH9 at a final concentration of 0.063 µg/ml. No PrPd labelling (as indicated by the absence of brown staining) was observed in the tissue sections tested. Scale bars = 200 µm. (DOCX) [file pone.0091143.s003.docx]

**Figure S3: Comparative negative control tissue sections from sheep, goat, deer and cow stained with ROS-IH9**

Panel A shows the dorsal motor nuclei of the vagus nerve (DMNV) at the level of the obex from sheep. Panel B shows the DMNV at the level of the obex from goat. Panel C shows cerebellum from deer. Panel D shows spinal tract from cow. Tissues were obtained from animals known not to be infected with a TSE. All tissues sections were stained with ROS-IH9 at a final concentration of 0.063 µg/ml. No PrP^d^ labelling (as indicated by the absence of brown staining) was observed in the tissue sections tested. Scale bars = 200 µm.
