# Supplementary material for: Prion Protein-Specific Antibodies that Detect Multiple TSE Agents with High Sensitivity
Source: PLoS One. 2014 Mar 7;9(3):e91143. doi: 10.1371/journal.pone.0091143 (PMC3946747; doi:10.1371/journal.pone.0091143)
Supplement: Table S1 — Immunisation strategy. (DOCX) [file pone.0091143.s004.docx]

**Table S1: Immunisation strategy**

| **Expt group** | **Mouse**  **ID** | **Adjuvant** | **Boost interval after 1^st^ immunisation (weeks)** | **Fusion ID** | **Resultant mAbs** |
| --- | --- | --- | --- | --- | --- |
| **1** | **0** | Titre Max | 4 | F779 | FH6, HC2, FA6, BE2 |
|  | **1** |  | 8 | F782* | BH1, BC6, HC7, JC4 |
|  | **2** |  | NA | F766 | CF5, EC9, IH11, IH9 |
| **2** | **0** | Quil A | 4 | F782* | (as above) |
|  | **1** |  | 6 | F776 | BD12, DB12, BA6, DC12 |
|  | **2** |  | 8 | F772 | FH10, HB4, IF1, FD1, |
| **3** | **0** | Alum | 4 | F773 | DE3, BF5, JB10, HF9 |
|  | **1** |  | NA | F763 | AE11, EG6, FD12, EA6 |
|  | **2** |  | 8 | F782* | (as above) |

*Fusion F782 was created by pooling cell suspensions of spleens from the mice indicated.

Table S1 illustrates the immunisation strategy used to generate the monoclonal antibodies. Different adjuvants and boosting regimes were used depending on the individual mouse. NA indicates that mice were not given a boost after first immunisation. The nomenclature of the antibody corresponds to the plate identification, row and well number from which the best responders were selected from for cloning e.g. ROS-AE11, plate A, row E, well number 11.
