# Supplementary material for: Prion Protein-Specific Antibodies that Detect Multiple TSE Agents with High Sensitivity
Source: PLoS One. 2014 Mar 7;9(3):e91143. doi: 10.1371/journal.pone.0091143 (PMC3946747; doi:10.1371/journal.pone.0091143)
Supplement: Table S2 — Details of ruminant species used to assess five ROS- antibodies by Western blotting (WB) and immunohistochemistry (IHC). (DOCX) [file pone.0091143.s005.docx]

**Table S2. Details of ruminant species used to assess five ROS- antibodies by Western blotting (WB) and immunohistochemistry (IHC)**

| **Animal ID** | **Species** | **Genotype** | **TSE agent** | **Exposure** | **Age*** | **Status** |
| --- | --- | --- | --- | --- | --- | --- |
| HD46 (IHC) | Sheep | VRQ/VRQ | Classical scrapie | Oral | 6 | Clinical |
| I942 (WB) | Sheep | VRQ/VRQ | Classical scrapie | Natural | 48 | Clinical |
| G08-1131 (both) | Goat | I142M | Classical scrapie | Natural | 41 | Preclinical |
| J3011 (IHC) | Sheep | AHQ/AHQ | CH1641 scrapie | Intracerebral | 25 | Clinical |
| 41x28 (WB) | Sheep | ARQ/AHQ | CH1641 scrapie | Intracerebral | 60 | Clinical |
| SVS442/90 (IHC) | Cattle | - | C-BSE | Natural | 58 | Clinical |
| BBB/4/5 (WB) | Cattle | - | C-BSE | Natural | NR† | Clinical |
| L283 (IHC) | Sheep | ARQ/ARQ** | Cattle C-BSE | Oral | 29 | Clinical |
| N232 (WB) | Sheep | ARQ/ARQ** | Cattle C-BSE | Oral | 20.6 | Clinical |
| S12694 (both) | Goat | I142I | Goat C-BSE | Oral | 26 | Clinical |
| 009 (both) | Red Deer | Q226Q | Cattle C-BSE | Oral | 58 | Clinical |
| 06-1825 (IHC) | Sheep | AHQ/AHQ | Atypical scrapie | Intracerebral | 31 | Clinical |
| M278 (WB) | Sheep | ARQ/ARQ*** | Atypical scrapie | Natural | 36 | Clinical |

(both) indicates that tissue from the same animal was used for IHC and WB. NR† – not recorded here as tissue was a macerate prepared form multiple field cases *In months, age for natural infections or time post-challenge for experimental infections. **LL and ***FF at codon 141
