# Supplementary material for: Prion Protein-Specific Antibodies that Detect Multiple TSE Agents with High Sensitivity
Source: PLoS One. 2014 Mar 7;9(3):e91143. doi: 10.1371/journal.pone.0091143 (PMC3946747; doi:10.1371/journal.pone.0091143)
Supplement: Table S3 — Comparative analysis of subjective scoring for PrPd using five different ROS- antibodies in TSE-affected ruminants. (DOCX) [file pone.0091143.s006.docx]

**Table S3. Comparative analysis of subjective scoring for PrP^d^ using five different ROS- antibodies in TSE-affected ruminants**

| **Antibody** | **ROS-IH9** | **ROS-BH1** | **ROS-BC6** | **ROS-FH6** | **ROS-FH10** |
| --- | --- | --- | --- | --- | --- |
| **Conc [µg/ml]** | **0.063** | **0.134** | **2.27** | **0.093** | **20.8** |
| **TSE agent / species** |  |  |  |  |  |
| Scrapie / sheep | 2.5 | 2 | 2.5 | 2 | 1.5 |
| Scrapie / goat | 2 | 2 | 2 | 2.5 | 2 |
| CH1641 / scrapie | 1.5 | 0 | 0 | 0 | 0 |
| BSE / cattle | 2.5 | 3 | 1.5 | 3 | NT |
| BSE / sheep | 2.5 | 3 | 3 | 2.5 | 1.5 |
| BSE / goat | 3 | 3 | 2.5 | 2.5 | NT |
| BSE / deer | 1.5 | 2 | 2 | 1.5 | 1 |
| Atypical scrapie / sheep | 3 | 0 | 0 | 1 | 1.5 |

Scores vary between 0 (absence of PrP^d^ labelling) to 3 (strongest labelling). Working dilutions for each antibody are given in μg/ml. NT means not tested.
