# Supplementary material for: Prion Protein-Specific Antibodies that Detect Multiple TSE Agents with High Sensitivity
Source: PLoS One. 2014 Mar 7;9(3):e91143. doi: 10.1371/journal.pone.0091143 (PMC3946747; doi:10.1371/journal.pone.0091143)
Supplement: Table S4 — Arbitrary scoring of PrPd in ME7 and 87V scrapie, using ROS-BH1 and 6H4. (DOCX) [file pone.0091143.s007.docx]

**Table S4: Arbitrary scoring of PrP^d^ in ME7 and 87V scrapie, using ROS-BH1 and 6H4**

| **TSE agent** | **ME7** | | **87V** | |
| --- | --- | --- | --- | --- |
| **Antibody** | **ROS-BH1** | **6H4** | **ROS-BH1** | **6H4** |
| **Conc [µg/ml]** | **0.2** | **3.0** | **0.2** | **3.0** |
| **Brain region** |  | | | |
| Cortex (caudate) | 1 | 0 to 1 | 2 to 3 | 2 |
| Cortex (hippocampus) | 1 to 2 | 0 to 1 | 2 | 1 to 2 |
| Hippocampus | 2 | 2 | 1 to 2 | 1 |
| Thalamus | 2 | 2 | 3 | 2 to 3 |
| Hypothalamus | 1 to 2 | 1 | 2 | 1 |
| Colliculous | 1 to 2 | 1 | 3 | 2 |
| Brain stem | 3 | 3 | 3 | 2 to 3 |
| Cerebellum | 1 | 1 | 1 to 2 | 1 to 2 |

Scores vary between 0 (absence of PrP^d^ labelling) to 3 (strongest labelling). Working dilutions for each antibody are given in μg/ml.
